# Barriers and facilitators to linkage to ART in primary care: a qualitative study of patients and providers in Blantyre, Malawi

**DOI:** 10.7448/IAS.15.2.18020

**Published:** 2012-12-31

**Authors:** Peter MacPherson, Eleanor E MacPherson, Daniel Mwale, Stephen Bertel Squire, Simon D Makombe, Elizabeth L Corbett, David G Lalloo, Nicola Desmond

**Affiliations:** 1 Clinical Group, Liverpool School of Tropical Medicine, Pembroke Place, Liverpool, UK; 2 TB and HIV Group, Malawi–Liverpool–Wellcome Trust Clinical Research Programme, Blantyre, Malawi; 3 International Health Group, Liverpool School of Tropical Medicine, Pembroke Place, Liverpool, UK; 4 HIV & AIDS Unit, Ministry of Health, Lilongwe, Malawi; 5 Department of Clinical Research, London School of Hygiene and Tropical Medicine, London, UK

**Keywords:** HIV testing and counselling, antiretroviral therapy, linkage to care, qualitative studies, sub-Saharan Africa

## Abstract

**Introduction:**

Linkage from HIV testing and counselling (HTC) to initiation of antiretroviral therapy (ART) is suboptimal in many national programmes in sub-Saharan Africa, leading to delayed initiation of ART and increased risk of death. Reasons for failure of linkage are poorly understood.

**Methods:**

Semi-structured qualitative interviews were undertaken with health providers and HIV-positive primary care patients as part of a prospective cohort study at primary health centres in Blantyre, Malawi. Patients successful and unsuccessful in linking to ART were included.

**Results:**

Progression through the HIV care pathway was strongly influenced by socio-cultural norms, particularly around the perceived need to regain respect lost during a period of visibly declining health. Capacity to call upon the support of networks of families, friends and employers was a key determinant of successful progression. Over-busy clinics, non-functioning laboratories and unsuitable tools used for ART eligibility assessment (WHO clinical staging system and centralized CD4 count measurement) were important health systems determinants of drop-out.

**Conclusions:**

Key interventions that could rapidly improve linkage include guarantee of same-day, same-clinic ART eligibility assessments; utilization of the support offered by peer-groups and community health workers; and integration of HTC and ART programmes.

## Introduction

Despite being one of the poorest countries in the world [1], with an HIV prevalence of 11% [1], Malawi has made impressive achievements in scaling up provision of HIV testing and counselling (HTC) and antiretroviral therapy (ART). By June 2011, over 3 million Malawians had undergone HTC since programme inception and nearly 400,000 HIV-positive individuals had initiated ART [2]. Malawi pioneered the “public health approach” to ART, including decentralization of services to primary care level, a limited number of treatment regimens and laboratory investigations and standardized facility- and district-level reporting [3,4].

Rapid scale up of HTC and ART delivery has now been achieved in a number of sub-Saharan African countries using the public health approach [5,6]. However, coverage of ART in the sub-Saharan African region still remains suboptimal and Joint United Nations Programme on HIV/AIDS (UNAIDS) estimates that only 37% of people in need of ART had initiated treatment by 2009 [7].

**Figure 1:**
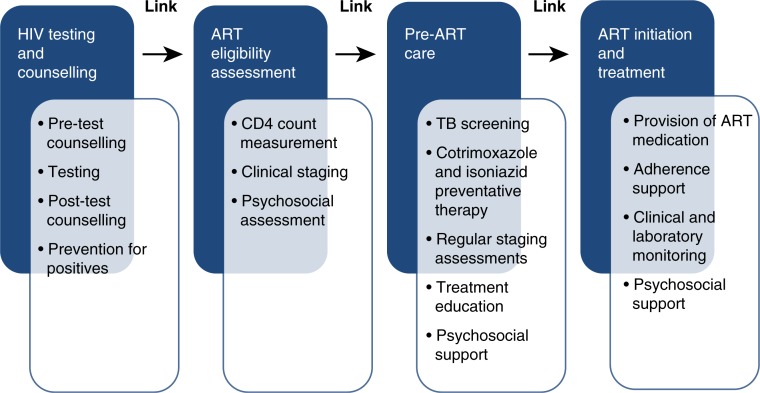
The HIV care pathway and services provided at each step.

Successful initiation of ART involves progression along a number of steps on the HIV care pathway, which links HTC to initiation of ART [8]. The HIV care pathway (Figure 1) entails successful completion of HTC, ART eligibility assessments (WHO clinical staging with or without CD4 count measurement), pre-ART care (including ART education and identification of a treatment guardian) and initiation of ART and subsequent adherence to treatment. Failure to link between any of these steps may result in a delay in treatment initiation or a drop-out of care. Because most HTC and ART programmes in sub-Saharan Africa have developed in parallel, and patient progress is not tracked or reported on, there are large gaps in understanding patient flow [9]. Drop-out and delayed linkage are likely to be significant contributors to the suboptimal population coverage of ART [10], and mathematical modelling suggests that improved rates of linkage could reduce HIV associated deaths and contribute to reductions in new infections [11].

A systematic review of HIV linkage [10] identified only two prospective cohort studies [12,13], both from hospital settings in South Africa. The authors estimated that only one-fifth of patients are retained in care continuously and go on to initiate ART [10]. They concluded by calling for more data from a wider range of settings to help understand the reasons for high rates of drop-out.

We conducted a qualitative study with patients and providers to better understand the patient, provider and health service barriers and facilitators contributing to progression through the HIV care pathway.

## Methods

### Study design

This was a qualitative study nested within a cohort study that investigated patient flow from HIV diagnosis to initiation of ART at primary care level. Baseline characteristics of cohort study participants, patterns of HTC and linkage to ART have previously been described [14,15].

### Study site and population

Two primary health centres (Ndirande Health Centre and Chilomoni Health Centre) were selected as study sites. At the commencement of the study, these clinics were the largest primary care providers of ART in Blantyre, with a combined total of 5720 individuals having initiated ART since ART became available in 2003 [2]. Similar to many national ART programmes in sub-Saharan Africa, ART delivery in Malawi has been decentralized to primary care centres. These two health centres were selected as they were felt to be representative of urban clinics offering HTC and ART in urban resource-limited settings in sub-Saharan Africa.

At the time of the study, under Malawian national HIV care guidelines, HTC was recommended for all individuals attending facilities, regardless of the presenting condition. Following HIV diagnosis, individuals underwent an eligibility assessment consisting initially of WHO clinical staging, with individuals in stage 3 or 4 referred directly for ART and individuals in stage 1 or 2 referred for CD4 count measurement. Upon completion of CD4 count measurement (which was conducted at the city's central hospital), individuals with a CD4 count of<250 cells/ul were also referred for ART initiation [16].

Chilomoni Health Centre is situated in the suburb of Chilomoni, which is located on the foothills of Mount Michiru. The suburb has little access to public services, such as piped water or sanitation, and subsistence farming is common. Ndirande Health Centre is located within the central market area of the urban slum of Ndirande. Ndirande is more densely populated than Chilomoni and supports a large amount of formal and informal trading. Both clinics offered HTC provided by counsellors trained by Ministry of Health, five days a week. At both clinics, there was a weekly ART clinic where ART initiation and clinical follow-up was provided by ART trained nurses. In addition, both clinics had a weekly psychosocial patient support and education group for patients awaiting ART initiation.

### Study participants

Between January and April 2011, newly diagnosed HIV-positive adults (16 years or older) were recruited at Ndirande and Chilomoni Health Centres and followed up for six months to assess their completion of steps on the HIV care pathway. From July to September 2011, we purposively sampled participants from this group of patients and invited them to participate in the qualitative study. Two groups of patients were sampled: those who had successfully linked from HIV diagnosis to ART initiation during six months of follow-up, and those who had not. A research assistant working in the study clinics identified potential participants by reviewing study files to determine whether ART had been initiated during the six-month follow-up.

From previous studies in South Africa and Malawi [13,17], we anticipated that gender, age and poverty might be important factors in determining progression through the HIV care pathway. In the parent cohort study [15], poverty was defined using a household proxy means wealth-ranking approach [18]. Therefore, we selected patients with a range of characteristics, including men, women and pregnant women; older and younger people; and people who were classified as coming from poorer and wealthier households. We set a minimum number of participants for inclusion (15 ART initiators and 15 individuals who did not initiate ART). Participants were recruited until saturation of data was achieved.

In addition, we interviewed health workers who provided HTC, pre-ART and ART services at the two clinics. Two cadres of health workers were recruited: counsellors (who undertook HTC and provided pre-treatment psychosocial support and education); and nurses, midwifes and clinical officers (who undertook ART eligibility assessments and initiated ART). We set out to interview a minimum of five health workers from each clinic.

### Data collection and analysis

A Malawian social science research assistant received intensive training on conducting sensitive qualitative interviews. Semi-structured interviews were conducted in Chichewa, the local language, and took place either in the health facility or in the participant's residence. Interview topic guides were developed collaboratively by the study team and incorporated themes on potential barriers and facilitators to linkage to ART. These themes were developed from reviews of the existing literature on care seeking for HIV and other illnesses (such as tuberculosis). At a weekly meeting, the topic guides were reviewed by the investigators and the research assistant and modified to incorporate emerging themes. Topic guides were translated into Chichewa and translated back into English by a Chichewa speaker who was not involved in the study.

All interviews were audio-recorded by the research assistant at a private location of the participants’ choosing (either at the clinic or at home). Interviews were transcribed into Chichewa scripts at a dedicated qualitative data centre. A third research assistant who had not transcribed the original audio file undertook translation into English. A sample of 5% of English transcripts were translated back into Chichewa and reviewed for consistency of translation.

Two researchers (PM & EEM) undertook qualitative analysis using a framework approach [19]. Each researcher independently coded initial samples of transcripts and the results were compared to assess the validity of the framework and to identity further themes for inclusion in the coding system. Following modification of the framework, coding was undertaken collaboratively. Analysis involved thematic charting, which encourages qualitative researchers to identify and summarize common themes arising from the data [19]. Data coding and analysis of themes were undertaken using the NVIVO-9 software platform (QSR, Melbourne, Australia). To aid conceptualization of barriers and facilitators to ART, a socio-ecological conceptual framework was developed [20,21].

### Ethical considerations

The College of Medicine of Malawi Research Ethics Committee and Liverpool School of Tropical Medicine granted ethical approval for the study. All participants gave written informed consent.

## Results

### Baseline characteristics

Between May and September 2011, 30 newly diagnosed HIV-positive adults were recruited to the study and participated in semi-structured interviews (15 from Chilomoni and 15 from Ndirande). Thirteen participants were men and 17 women, of whom 10 were pregnant (Table 1). Participants’ ages ranged from 18 to 45 years. In total, 15 participants had successfully initiated ART during the six-month follow-up, whilst the remaining 15 participants had not initiated ART.

**Table 1:** Summary of participants

Method	Participant type	Gender	Age range
Semi-structured interviews	Individuals who initiated ART	6 Men	25–41 years
		9 Women (6 ANC)	19–37 years
	Individuals who did not initiate ART	7 Men	21–45 years
		8 Women (4 ANC)	18–31 years
	Nurses & clinical officers	2 Men	27–33 years
		3 Women	26–35 years
	Counsellors	1 Men	25 years
		4 Women	23–41 years

ANC: Participant was pregnant and attended the antenatal clinic.

Ten healthcare workers were also recruited to the study and participated in in-depth interviews. Of these, five were counsellors, three were nurses and two were clinical officers.

### Conceptualizing determinants of drop-out and delay

**Figure 2:**
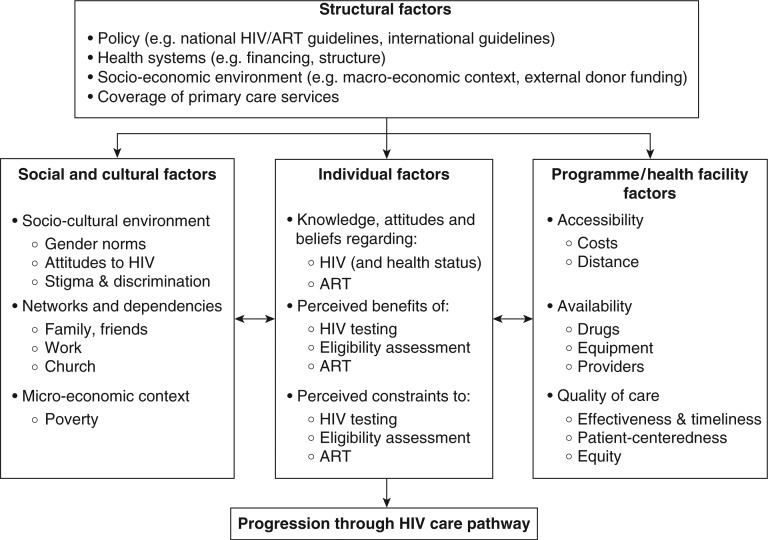
Socio-ecological conceptual framework of barriers and facilitators of progression through HIV care pathway. Based on a framework developed by Roura et al. [21].

Thematic analysis identified key determinants of drop-out and delay at the individual-, socio-cultural-, programmatic/policy- and structural levels (Figure 2). Participants who did not initiate ART experienced negative effects from factors at all of these levels, whereas participants successful in initiating treatment were frequently able to utilize factors from one or more of these levels to their benefit. Failure of linkage was then multifactorial and negative factors at more than one level frequently interacted, having a multiplicative effect on the risk of drop-out. For example, participants who were poor (and so less able to cope with the expense of clinic visits) and who were not able to draw upon support from networks of friends and families reported greater difficulty in staying in care than participants with only one of these factors. All patients and providers were affected to some degree by the structure, organization and limitations of the Malawian public healthcare system and by the national HIV care policies that were in place, although individual behaviour in response to this environment varied considerably.

### Linkage to ART

#### Decision to undergo HTC

The first step on the HIV care pathway is the individuals’ decision to undergo HTC, and this was strongly influenced on one hand by their awareness of their ailing health and their knowledge of the potential benefits of ART treatment, and on the other by their fear of the consequences of being diagnosed HIV positive. An individual's decision to attend the clinic to undergo HTC was made independently and was not discussed with partners, family members or friends.

The individuals’ principal motivation to attend the clinic and request HTC was a prolonged period of declining health that impacted upon their ability to work or carry out household chores (Table 2, quote 1.1). Therefore, individuals saw HTC as the first step towards regaining strength and avoiding an impending catastrophic social event, such as loss of employment or abandonment by a partner (Table 2, quote 2.2). Men also worried that, with increasingly outwardly obvious signs of failing health, they would be perceived to be less masculine and less respectable, with the assumption made that they could not provide for their family and not able to perform sexually (Table 2, quote 2.1).

**Table 2:** Selected themes and quotes from providers and patients

Theme	Quote
1. HTC followed a period of prolonged and outwardly visible declining health and was sought independently and in private	1.1. “I am one of the people who does hard jobs: I stay in the sun the whole day building houses. From last year I was having stomach pains often, so I was just taking painkillers. Then I reached a stage of having fever regularly. So I was working one day and I felt weak and dizzy. I had heard that near my house there is VCT so I decided I have to go for a test.” Male participant, Chilomoni, 39 years, initiated ART.
2. Prevalent gender norms strongly influenced care-seeking	2.1. “I was a person and I had a good looking body. Even when I was walking people knew that aah that man that is passing there, he is really a man. […] So I was examining myself and could see that my body was not all right. That is what made me think that aah it's better to go where I hear that they do some tests—maybe I have a disease. That is why I mustered up boldness to go for testing.” Male participant, Ndirande, 25 years, did not initiate ART.
	2.2. “Have you seen men doing household chores like washing dishes [laughs]? In the morning, when the husband wakes up, you have to give him water to bathe – and he comes in the night! […] We have responsibility of taking care of the children so we say ‘aah if I die, will my husband take care of my children?’ They have the freedom to have another wife, taking the children to that other woman. That's why we say ‘let us go to the hospital!’” Female participant, Ndirande, 32 years, initiated ART.
3. Provider-initiated HTC in ANC was highly acceptable	3.1. “[At the antenatal clinic] everyone was told that she is supposed to do the test. They test everyone for the disease of what do you call it, HIV? So when I was told that you should have a test I didn't worry about what was going to happen. I was already knowing that it was there and so I knew when I went there it would not be a difficult thing.” Pregnant women, Ndirande, 30 years, did not initiate ART.
	3.2. “As of now, we give the first priority [for testing] to a woman who is pregnant. If the woman is expectant, she is expecting a baby … we want to protect the unborn child so that they should be born free from the virus.” Counsellor, female, Ndirande, 23 years.
4. ART eligibility assessments were inappropriate for primary care and failure to complete assessments was a major reason for drop-out	4.1. “Sometimes we have more than 80 clients, so out of that number, to do WHO staging for everybody, it seems as if we are delaying others. So we just tell them in a short-cut way and refer them for CD4 count to ensure that everybody should feel that they have been helped in the right time.” Nurse, female, Ndirande ANC, 35 years.
	4.2. “You see that the person is weak when he is entering into the room and you already know. Yes, we say that you shouldn't judge a book by its cover, but somehow you are able to see the way it is.” Nurse, male, Ndirande, 33 years.
	4.3. “I went there [Central Hospital] and they said ‘The machines are not working’. Another time, I went and reached the point that I did a blood test. After that they told me ‘Come on Tuesday and hear your results’. On Tuesday I begged the transport money from my boss. When they looked at the result they said ‘that blood which was taken that time … the machine was not working. You will have to come again’.” Male participant, Ndirande, 41 years, initiated ART.
5. Networks of family members, friends and employers were important in supporting progression through the HIV care pathway and retention in care	5.1. “My friend who I am working with, she encourages me because she is taking the treatment. She tells me ‘do you see how I am looking compared to the way I was looking in the past? Is it the same? You have to start receiving the treatment so you can look the way I am.’” Female participant, Chilomoni, 29 years, initiated ART.
	5.2. “My family and my relatives they all agree because they have seen other people reach the point of being finished [died]. But when they started receiving this treatment they recovered and their bodies become as it was before. So they are the ones who encourage me the most.” Female participant, Chilomoni, 29 years, initiated ART.
	5.3. “Talking of the job, I can say that it helps me. I still go to work there and they helped me [gave money] to go to the clinic so that I could receive the treatment that I needed.” Male participant, Ndirande, 41 years, initiated ART.
	5.4. “That time we were living far from the clinic […]. But because of my wish to receive the treatment, I wanted to attend. I was borrowing money from friends. If I thought they would give me money, I would have to ask three days before the journey so that I would know if they would give me the money. Eeh these are big problems if you are far from the clinic.” Male participant, Ndirande, 31 years, did not initiate ART.
	5.5. “I am not married, so I do things myself, like cooking on my own. […] I do part-time work, gardening and landscaping in lots of different areas. So if I go to the clinic, when will I come back? Will I eat?” Male participant, Chilomoni, 21 years, didn't initiate ART.
6. Pre-ART care infantilized patients and made them dependent on others	6.1. “The things they tell you not to do, like do not eat certain foods and drinks like beer … and cigarettes—they say ‘do not smoke cigarettes’. Maybe you like drinking beer, but they say that you should stop. You have to stop it.” Male participant, Ndirande, 25 years, did not initiate ART.
	6.2. “So they gave me advice that I should avoid doing childish things like that. If I do, I should use condoms. But we are not youths we are elderly people.” Male participant, Ndirande, 45 years, did not initiate ART.
	6.3. “If you do not bring your guardian when you are learning you are sent back. They say ‘No. Go and get your guardian and bring her here.’ So, if I did not bring my guardian here, I would not be given the treatment.” Female participant, Ndirande, 19 years, initiated ART.
7. Health workers perceived themselves to be gatekeepers in the HIV care pathway, determining who should be permitted to access treatment according to their needs and deservingness	7.1. “But I have done the test and I have been coming here at the clinic telling the doctor that I am sick. The doctor just gives me Panadol or aspirin, even LA [malaria treatment]. You will just receive it and off you go home not knowing what to do.” Female participant, Chilomoni, 30 years, did not initiate ART.
	7.2. “On this point we make very strict procedures because these medicines are not commonly found. We look at the client's personal life and their problems and we see that if we ignore these problems, we will kill the client [by giving them ART].” Nurse, female, Ndirande, 26 years.
	7.3. “So we first see how these people look physically. Maybe that patient is very weak and he has no power. So we say that it is good that he should start [ART] quickly and he can be shifted [up the list.]” Nurse, female, Chilomoni, 26 years.

Delay in attendance for HTC was strongly influenced by socio-cultural norms, particularly gender norms and conceptualizations of the consequences of HIV diagnosis. Emphasizing the private manner in which a decision to test was made, many participants described this period in terms of a private struggle between their fears of the consequences of not testing (abandonment, poverty, illness and death) and their fears of the consequences of HIV diagnosis (stigma and loss of status) (Table 2, quote 2.1). Participants reported being spurred into action to test once their health deteriorated to the point they felt that others might identify their HIV status. By doing so, they attempted to regain control over who had knowledge of their HIV status and the manner in which it was disclosed.

Contrary to men and non-pregnant women who made independent decisions to attend HTC, pregnant women had a very different journey to HTC. Most of the pregnant women interviewed stated that they did not have symptoms that led them to suspect they might be living with HIV. In accordance with national HIV guidelines, midwives offered HTC to all women attending the antenatal clinics (ANCs). The perception of the pregnant women interviewed was that there was little opportunity to refuse HTC as health workers were seen as powerful, senior members of the community. There was also a perception that the government had decreed that all pregnant women should be tested for HIV, and this was interpreted to mean that there was no opportunity to refuse testing (Table 2, quote 3.1).

Counsellors working in the ANC held the view that HTC was mandatory for pregnant women and that the Ministry of Health had promoted this policy. They were supportive of the provider-initiated HTC approach, reasoning that it would help prevent mother-to-child transmission of HIV (Table 2, quote 3.2). However, clinicians working outwith the ANC in the general outpatients department, although recognising the value of HTC, took a much less proactive approach towards HIV testing. They limited their requests for testing to individuals in whom they had a high clinical suspicion of HIV, meaning that provider-initiated HTC was not fully implemented outside of the ANCs.

#### Eligibility assessments

Following completion of HTC, the counsellor who had performed the test referred the patient to a clinician for ART eligibility assessment. Participants reported that this system meant that they would start the day at the clinic by queuing to see the clinician for management of their presenting ailment (or at the antenatal clinic, if pregnant), then queue to see the counsellor for HTC and finally (often late in the day), queue again to see the clinician for ART eligibility assessment. Eligibility assessment initially involved the clinician performing WHO clinical staging followed by a referral for measurement of CD4 count for patients in WHO stage 1 or 2.

WHO staging was frequently not completed on the same day as diagnosis of HIV infection. If it was late in the day, or the clinician had left the clinic (absenteeism was commonly reported by patients but not by health workers), patients were advised to return on another day. Participants frequently described making repeat visits to the clinic before being WHO staged, or dropping out of care at this point as they could no longer manage to keep returning to the clinic due to pressures of time, employment and expense.

Even when the clinician was available to complete eligibility assessments, there were considerable problems with health workers’ and participants’ completion of WHO clinical staging. Clinicians reported that the process of staging was difficult and time-consuming (Table 2, quote 4.1). If they suspected a complex clinical problem (that could signify a WHO stage 3 or 4 illness), they often found that the staging condition could not be confirmed due to lack of diagnostic laboratory or radiological services at the primary healthcare centre. Instead of formally WHO staging patients according to guidelines, clinicians reported that they would often base their eligibility assessment on a superficial assessment of the patient's wellbeing (Table 2, quote 4.2).

If patients were referred for CD4 count measurement to complete their ART eligibility assessment, this was a particularly common point of drop-out from the HIV care pathway. At the time of this study, CD4 counts could only be performed at the city's central hospital and required at least two visits to complete measurement (one to give blood and the second to collect the result). The CD4 count machine was frequently out of service and results were misplaced or lost, necessitating repeat visits (Table 2, quote 4.3). Each journey for CD4 measurement would require substantial expenditure, often borrowed from family members or friends.

#### Pre-ART care

In contrast to the private, independent role taken when accessing HTC, participants described adopting a dependent care-seeking role following completion of ART eligibility assessments. In particular, they found themselves relying on the financial, emotional and practical support of networks of family members (for example, to provide money and childcare for clinic attendances), friends (especially members of their church congregations and friends who were also HIV positive or who were already taking ART) and employers (to provide time off work and money) (Table 2, quotes 5.1, 5.2 and 5.3). The process of adopting a dependent care-seeking role necessitated that participants disclose their HIV status to partners, family members and friends from whom they required support. There was a range of responses and reactions to the need for disclosure that did not appear to be related to any social or demographic characteristics of participants. Some participants reported that they found this process easy and that they valued the support received from others. Some participants reported that they were not keen to disclose their HIV status. However, they felt obliged to by the need to obtain money to attend the clinic for pre-ART care and to identify a treatment supporter.

This enforced dependent role extended to numerous facets of participants’ lives. In post-test counselling sessions, they were advised as to what food they should eat, to whom they should disclose their status (with most being advised to disclose to a family member who would act as their “treatment guardian”) and even how they should have sexual relationships. These were perceived as a set of rules that were imposed by health workers and which, if not strictly followed, would result in ART being withheld. Participants, especially older adults, felt that these rules infantilized them. This perception was reinforced by the fact that they had to attend pre-ART lessons accompanied by a relative, bringing back memories of attending school as a child (Table 2, quotes 6.1, 6.2 and 6.3).

Participants found that their interactions with the health facilities and providers in the pre-ART period had a major bearing on their ability to initiate ART, and their ability and willingness to remain in care if not yet ART eligible. Providers were often perceived to be rushed and unapproachable and did not explain clearly what was provided in pre-ART care or when and how they should initiate ART (Table 2, quote 7.1). Clinics were extremely busy and crowded, resulting in an unwelcoming environment and a disincentive to continued attendance.

For their part, health workers saw themselves as gatekeepers in the primary clinic system, determining who should and should not receive treatment according to national guidelines. ART was seen as a precious resource that required careful rationing to prevent misuse (Table 2, quote 7.2)

Study participants who had been successful in initiating ART reported that they experienced little difficulty in linking from pre-ART care to ART initiation. This could be because they had more advanced HIV infection and were prioritized by clinicians for expedited initiation onto ART. In both clinics, clinicians reported that an informal “monthly ART list” existed, limiting the number of patients who would be started on ART every month to avoid overburdening the ART clinic. Patients who clinicians perceived to be particularly in need of ART (usually patients with obvious outward signs of advanced HIV infection) were prioritized for the list, and clinicians discussed having to “squeeze” another patient onto the monthly list (Table 2, quote 7.3).

Participants who did not initially meet eligibility criteria, or who were not prioritised by clinicians for ART initiation, found it increasingly difficult to avoid dropping out of pre-ART care. They were less likely to have advanced HIV infection and self-identify themselves as urgently needing treatment. They saw little benefit from the time-consuming and expensive repeat clinic visits and the pressures of work and family commitments became their main priority. In addition, some participants reported that they felt that their continuous requests for support and money were having a negative impact on their relationships with family, friends and colleagues within the workplace (Table 2, quote 5.4 and 5.5).

## Discussion

The main finding from this study of barriers and facilitators to ART initiation was that delays and drop-out from the HIV care pathway in primary care were commonly reported by participants and strongly determined by factors at multiple levels. Figure 2 emphasizes that participants who did not initiate ART experienced negative effects from factors at all of these levels, whereas participants successful in initiating treatment were frequently able to utilize factors from one or more of these levels to their benefit. Individuals made private decisions to delay attending HTC because of fear of the consequences of their HIV-positive status being revealed. However, after diagnosis they became rapidly dependent on social support networks to navigate the ART eligibility assessment and pre-ART care periods. Improving upon the current low rates of linkage to ART [10] will require a comprehensive rethinking of how HIV care is delivered.

Of particular concern (and the point at which there was high reported drop-out) was the ART eligibility assessment period. Providers were overburdened by clinic workloads and felt unable to reliably complete WHO clinical staging assessments. Patients found the eligibility assessment period to be confusing and incurred substantial costs in making repeat facility visits. Completion of CD4 count was particularly problematic, requiring multiple facility visits and subject to laboratory failures and missing results. This closely mirrored findings from South Africa where low rates of CD4 completion led to failure of timely initiation of ART [22,23].

During the eligibility assessment period, patients relied on networks of support to borrow money, assist with childcare and allow time off work. A previous study from rural Malawi found that acceptance of ART by TB patients living with HIV was strongly influenced by their ability to afford transport to the hospital [24]. In a separate study, TB patients were reported to spend up to 574% of their monthly income during the pre-treatment period [25]. We recommend that national programmes should guarantee same-day, same-clinic eligibility assessments to newly diagnosed HIV-positive patients. To facilitate this, novel eligibility screening approaches will be required. In particular, simplified alternatives to the WHO clinical staging assessment may have to be developed for use in primary healthcare centres [14] and expansion of point of care CD4 count measurement systems [26] are required. Operational research is required to determine the best means of implementing and funding streamlined approaches to ART eligibility assessment [27].

Similar to the ART eligibility assessment period, in the pre-ART period, participants adopted a dependent care-seeking role, seeking help from networks of families and friends. Participants who were not able to draw upon these support networks or who felt unwilling to continue asking for support were at high risk of drop-out. Treatment supporters have been a key component of many public health ART programmes [28], and their use is associated with improved survival on ART [29]. Here we found that patients found it increasingly difficult to continue requesting support following the immediate post-diagnosis period. Previous studies from Namibia [30] and Malawi [31] have highlighted the “household shock” experienced when a member of the family is diagnosed HIV positive and this is manifested both economically and psychologically. This initial period of shock can extend into long-term hardship and exacerbate mutual feelings of guilt and resentment from both carers and patients, especially when ART is delayed [30]. Social tensions may be resolved through more family-orientated HIV clinics, where patients and carers can receive group psychosocial support and assistance to resolve difficulties together [32]. In addition, short-term social security cash transfers, such as the Disability Grant offered in South Africa, may help households overcome this acute crisis period and support access to ART, although care should be taken to avoid perpetuating further dependency [33].

In contrast to the highly dependent role adopted in the ART eligibility assessment and pre-ART periods, participants’ decision to attend the clinic and request HIV testing was made independently. Although validation of their decision was not sought from others before testing, their behaviour was strongly influenced by the anticipated response from partners and other community members. Anticipated stigma and ostracism are well-recognized barriers to the uptake of HIV testing [34] and are challenging to overcome when designing HIV-testing programmes [35]. We were encouraged to find that when provider-initiated HTC (PITC) was fully implemented, as in the ANC, it was highly acceptable both to patients and providers. Pregnant women were aware that HTC was offered to all women attending the ANC and seemed generally supportive of this approach. However, with the ANC, providers were less able to offer PITC due to pressures of over-busy clinics. They also saw less value in offering PITC, meaning that they became complicit in patients’ decisions to delay testing for HIV until it was unavoidable. Where ART is rapidly available and linkage rates are good, this could encourage providers to prioritize PITC [36].

Prevalent gender norms were found to be important determinants of both decisions to undergo HTC and of subsequent progression through the HIV care pathway. Widely held concepts of masculinity, where men were expected to portray an aura of respectability, financial success and sexual prowess, and femininity where women were expected to look after the household and raise the children, strongly inhibited individuals’ willingness to seek care. Previous studies of care-seeking patterns in tuberculosis suspects [37] and patients living with HIV and TB [38] have emphasized how these gendered attitudes influence delay and drop-out from care in sub-Saharan Africa. At the clinic-level, making services more responsive to the specific needs of men and women, (for example, having gender-specific peer groups within clinics and offering clinic-based support via mobile phone technology [39]) could reduce rates of drop-out by overcoming these barriers.

Health policy and health system structure were important factors in drop-out from the HIV care pathway. The separate HTC and ART registration systems located in different parts of the clinic subjected patients to long waits and multiple visits. A similarly over-complex pathway for diagnosis of TB contributed to previously high rates of drop-out and initial delay. The introduction of the DOTS approach considerably streamlined the TB care pathway and improved treatment outcomes [40]. A similar unified registration system, with one register that covered the entire HIV care pathway could cut down on unnecessary health worker visits, improve retention and would allow monitoring and evaluation of linkage outcomes.

Key strengths of this study were the use of qualitative methods and the inclusion of both patients’ and health workers’ voices. Nevertheless, social desirability may have biased participant and health worker responses. In particular, we found that health workers were understandably reluctant to overtly criticize the HIV care programme, limiting our ability to draw conclusions about how the structure of the programme influenced the care they provided. Although this study was conducted at two of the busiest primary care clinics in Malawi, it is possible that our findings may not be applicable to other settings that have differently structured health systems or socio-cultural environments. In particular, we noted that participants were reluctant to directly discuss their expectations for treatment and care following HIV diagnosis. The may reflect the Malawian context, where health workers are held in high esteem and their actions rarely questioned. Socio-cultural factors were important in determining linkages to ART. In other countries, with distinct socio-cultural traditions, the impact of support from networks of family and friends may differ.

## Conclusions

In this study, drop-out from the HIV care pathway was multifactorial. Lack of social networks, economic vulnerability and fear of the consequences of disclosure of HIV status are major contributors. At the programme level, overly busy clinics, inappropriate ART eligibility screening tests and unintegrated HTC and ART programmes resulted in failure of linkage. A key facilitator of linkage to ART was socio-economic support from networks of family members and friends. To rapidly improve linkage, we propose a rethink of the current HIV care model in primary care to include a guarantee of same-day, same-clinic eligibility assessments using point of care CD4 count measurement, greater utilization of the support offered by peer-support groups and community health workers, as well as an integration of HTC and ART services at the policy and programmatic levels.
